# Closing Sim2Real Gaps: A Versatile Development and Validation Platform for Autonomous Driving Stacks

**DOI:** 10.3390/s26041338

**Published:** 2026-02-19

**Authors:** J. Felipe Arango, Rodrigo Gutiérrez-Moreno, Pedro A. Revenga, Ángel Llamazares, Elena López-Guillén, Luis M. Bergasa

**Affiliations:** Electronics Department, University of Alcalá, 28805 Alcalá de Henares, Spain

**Keywords:** Sim2Real transfer, reality gap, performance gap, autonomous driving stack, development and validation platform (DVP), digital twin, parallel execution, real-world testing, CARLA simulator, robot operating system (ROS)

## Abstract

The successful transfer of autonomous driving stacks (ADS) from simulation to the real world faces two main challenges: the **Reality Gap (RG)**—mismatches between simulated and real behaviors—and the **Performance Gap (PG)**—differences between expected and achieved performance across domains. We propose a **Methodology for Closing Reality and Performance Gaps (MCRPG)**, a structured and iterative approach that jointly reduces RG and PG through parameter tuning, cross-domain metrics, and staged validation. MCRPG comprises three stages—**Digital Twin**, **Parallel Execution**, and **Real-World**—to progressively align ADS behavior and performance. To ground and validate the method, we present an open-source, cost-effective **Development and Validation Platform (DVP)** that integrates an ROS-based modular ADS with the CARLA simulator and a custom autonomous electric vehicle. We also introduce a two-level metric suite: (i) **Reality Alignment** via Maximum Normalized Cross-Correlation (MNCC) over multi-modal signals (e.g., ego kinematics, detections), and (ii) **Ego-Vehicle Performance** covering safety, comfort, and driving efficiency. Experiments in an urban scenario show convergence between simulated and real behavior and increasingly consistent performance across stages. Overall, MCRPG and DVP provide a replicable framework for robust, scalable, and accessible **Sim2Real** research in autonomous navigation techniques.

## 1. Introduction

Autonomous Vehicles (AVs) are a major focus of modern technological advancements. Current technology is laying the groundwork for a future where road travel can occur without human intervention. This innovation has attracted significant attention from various sectors—including industry, academia, society, and politics, involving researchers, car manufacturers, and tech companies. AVs offer extensive benefits, including improved road safety, enhanced energy efficiency, optimized traffic flow, and advancements in communication systems—such as the development of Vehicle-to-Everything (V2X) technologies. Two of the most important advantages of AVs in shaping the future of the automotive industry are: (i) the promise of significantly reducing road accidents by removing human error in vehicle control, and (ii) their significant potential to reduce traffic congestion as a crucial topic of future intelligent transportation systems [1,2,3].

The main technological challenges are related to safety issues during driving at Levels 4 and 5 defined by the Society of Automotive Engineers (SAE) [4], especially in areas not adapted for AVs—such as urban zones. In recent decades, numerous tests on AVs have been conducted worldwide to address these issues and accelerate their market introduction.

At this point, simulators play a pivotal role—particularly in addressing the crucial topic of Software (SW) validation. Implementing partially debugged algorithms directly in real-world vehicles can lead to serious consequences. To mitigate these risks, initial validations are traditionally performed using simulators, followed by real-world testing. A key challenge in this process lies in ensuring a smooth and reliable migration of algorithms from simulation to the real world, a process commonly known as Simulation-To-Reality (Sim2Real) transfer [5,6].

Testing in simulators mitigates the risk of accidents during development, ensuring algorithms are safer before they are tested in the real world. Furthermore, these Sim2Real tools offer a controlled environment in which we can easily replicate and test various driving conditions, enabling us to validate AVs in a wide range of scenarios. This includes corner cases and situations that would be challenging or hazardous to replicate in the real-world.

However, this process is only effective when the difference between the simulated and real behavior is minimal and well-defined, a concept known as Reality Gap (RG) [7]. RG refers to the discrepancy in ego-vehicle behavior between a simulated environment and the real world, caused by the inaccurate representation of real scenarios in simulation.

A strategy to minimize the RG is to employ high-fidelity driving simulators that stand out for their high level of similarity in the physical behavior of vehicles under various conditions—such as weather, road profile and surfaces, and lighting, among other factors. Nonetheless, there will always be discrepancies between simulated and real-world conditions that must be carefully handled to ensure AV reliability, keeping the RG within acceptable limits.

To address these discrepancies and further enhance the reliability of simulations, many platforms now integrate Digital Twin (DT)—a concept that is becoming increasingly significant each day. This technology involves creating a virtual replica of the real world, allowing the study and analysis of physical objects within a digital environment. When it comes to AVs, DT offers innovative solutions by providing a digital identity, synchronized visualization, and seamless interaction between simulated and real environments [8].

Building on the advantages offered by DT, a new methodology known as Parallel Execution (PE) has emerged, providing a direct and continuous interaction for model training and validation in the context of AVs. The PE approach—best illustrated in one of our previous works [9]—enables real-time synchronization between the ego-vehicle’s movements in the real world and its DT in simulation. The ego is mirrored in the simulator, and the synthetic data from the environment feeds the decision-making algorithm that controls the ego-vehicle. Moreover, PE serves as a crucial intermediate stage that bridges simulation and the real world, significantly reducing both time and resource requirements before performing validation in a fully real-world environment.

### 1.1. Motivation and Problem Statement

In this paper, we propose a methodology to iteratively close Sim2Real gaps. Discrepancies in ego-vehicle dynamics, sensor behavior, processing times, and interactions with agents and the environment introduce an RG that poses significant challenges when transitioning from simulation to real-world scenarios.

These differences directly affect the performance of the Autonomous Driving Stack (ADS), leading to behavioral variations between simulated and real-world tests. To address this issue, we introduce the concept of Performance Gap (PG)—defined as the discrepancy between the expected and actual performance of a system when transitioning from simulation to real-world environments. This gap arises not only from factors such as vehicle dynamics, sensor behavior, processing delays, and interactions with the environment—closely related to the RG—but also from the underlying Hardware (HW) infrastructure.

The choice between server computing and edge computing significantly impacts PG. Systems running on high-performance server computing benefit from greater processing power, lower computational constraints, and more efficient data handling, leading to optimized performance in simulations. However, in real-world deployments, autonomous systems often rely on edge computing, where computations occur on resource-limited devices closer to the data source. This shift can introduce latency, reduced processing capacity, and inconsistencies in performance, further widening the PG. Understanding and mitigating these HW related discrepancies is essential for improving the efficiency, reliability, and adaptability of autonomous systems in diverse operational conditions.

**Figure 1:**
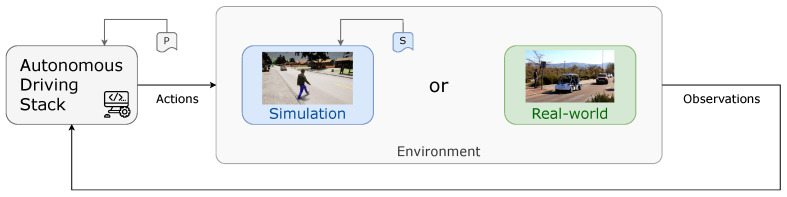
Autonomous driving stack workflow in simulation and real-world environments.

The first step in bridging performance gaps and enhancing an ADS is to study and understand how it interacts with both simulated and real-world environments—as illustrated in Figure 1—in a comprehensive, generic, and holistic manner. An ADS continuously generates actions and receives observations from its environment—either simulation or real-world—in a closed-loop process.

Actions refer to any type of output produced by the ADS—such as linear and angular velocity commands or local trajectory (waypoints). Observations, on the other hand, represent any data collected from the environment via sensors—including positions, steering-wheel angles, lateral deviations, object detections, and more. By comparing system behavior across both domains, researchers can better identify shortcomings, validate algorithms, and refine performance in real-world driving conditions.

**Figure 2:**
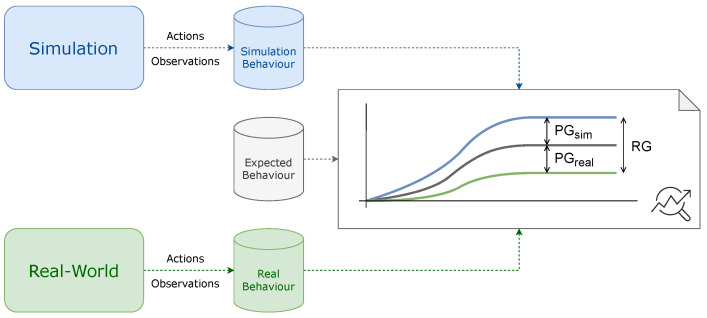
Sim2Real Gaps: Reality and performance gaps in both simulation and the real world.

Figure 2 illustrates how data from the ADS’s behavior in both simulation and real-world settings can be used to identify and analyze the two key issues: RG and PG. The corresponding actions and observations can be recorded for offline analysis. By holistically examining the logs generated in simulations and real-world tests, it is possible to identify these gaps and address their challenges, ultimately enhancing the performance of the ADS.

For example, urban environments with complex traffic dynamics, diverse weather conditions, and unpredictable obstacles present scenarios where the ADS’s performance is not optimized to address these new use cases. Moreover, simulation tools often fail to accurately replicate such real-world behaviors. These divergences significantly reduce the system’s ability to make robust, real-time decisions, thereby compromising its reliability and safety.

To address these gaps holistically, we defined two categories of parameters, as illustrated in Figure 1: the ADS’s Configuration Parameters (*P*) and the Simulator’s Setting Parameters (*S*). *P* are intrinsic to the ADS and include settings like sampling rate, measurement errors, detection thresholds, and more. Additionally, *S* govern the simulation environment and include factors—such as synchronous mode, frame rate, ego mass, sensor noises, and more.

By jointly optimizing these parameters—*P* and *S*—we can systematically address both gaps, enabling a more reliable and robust deployment of ADS across both simulation and real-world scenarios. For that reason, one of the main objectives in implementing an ADS lies in developing robust platforms and validation methods to ensure thorough algorithm debugging. Given the diversity of scenarios which ADS will face during the validation phase, the use of validation platforms is becoming crucial to ensure detailed and reliable validations.

### 1.2. Contributions

To address the aforementioned issue, we propose a comprehensive methodology designed to systematically reduce both the Reality Gap (RG) and Performance Gap (PG) when transferring autonomous driving stacks from simulation to real-world environments. In summary, the main contributions of this paper are listed as follows:

- Conceptual framing of Sim2Real gaps for ADS. We analyze and relate the RG and the PG for autonomous driving stacks.
- We propose a **Methodology for Closing Reality and Performance Gaps (MCRPG)**; this approach uses observations and metrics analysis with parameter fine-tuning to align behaviors across domains and to maximize ADS performance while progressively reducing both gaps, across three iterative stages (Digital Twin, Parallel Execution, and Real-World).
- We design and implement an open-source, cost-effective and robust **Development and Validation Platform (DVP)** that integrates CARLA, a real proving ground, and a vehicle through the Robot Operating System (ROS). The platform includes a built-from-scratch ADS and a fully developed real vehicle, enabling seamless switching among three iterative stages: Digital Twin, Parallel Execution, and Real-World.
- We define a metric suite for gap reduction based on two-level evaluation: (i) **Reality-Alignment** via Maximum Normalized Cross-Correlation (MNCC) between different signals (poses, steering, velocity, detections), and (ii) **Ego-Vehicle Performance** via safety, comfort and driving efficiency metrics.
- Through empirical validation across three iterative stages—Digital Twin, Parallel Execution, and Real-World—we demonstrate a progressive convergence between simulated and actual behavior, with performance indicators becoming increasingly consistent across stages and iterations.
- Beyond the methodology, we document a modular ADS and HW platform build that other groups can replicate or extend, fostering accessible Sim2Real research.

### 1.3. Paper Structure

The remainder of the paper is organized as follows. Section 2 reviews related efforts on Sim2Real for AVs and motivates the need for a holistic, system-level approach. Section 3 presents MCRPG, detailing the Digital Twin, Parallel Execution and Real-World stages, the action/observation flow, and the tuning process of *P* and *S* parameters. Section 4 describes the DVP: the ROS-based ADS, the CARLA simulation framework and the TABBY EVO HW platform. Section 5 specifies the experimental setup: the scenario, the metric suite (reality-alignment and ego-performance), and reports results across iterative loops and stages. Finally, Section 6 concludes with key findings, limitations, and future lines—including broader scenarios, richer metrics, cross-platform validation, and automated tuning.

## 2. Related Work

Research on bridging the Sim2Real gaps for AVs has made significant progress, focusing on improving simulation fidelity and validation processes. Various studies have explored innovative methods to minimize discrepancies between simulated and real environments, emphasizing simulator optimization, leveraging digital twin, and refining testing frameworks.

Stocco et al. [10] addressed the challenge of generalizing testing results obtained in driving simulators to physical platforms by characterizing the RG in the context of AVs. They propose using small-scale vehicles as an intermediate step to evaluate AV performance before full-scale testing, which is often costly and hazardous. Using the Donkey Car platform, the study conducts a comparative analysis between virtual and real-world environments, assessing the performance of three state-of-the-art lane-keeping models through metrics—such as steering angle, lateral deviation, and predictive uncertainty. This work stands out as one of the first comprehensive studies comparing simulated and real-world testing results, demonstrating the transferability of driving behavior across environments and providing a safe and accessible approach for research in this domain.

Pasios and Nikolaidis [11] focused on enhancing the visual realism of simulation environments by proposing a photorealism framework for the CARLA simulator. Their approach introduces a novel image-to-image translation method that processes intermediate information, known as Geometry Buffers (G-Buffers), from the rendering pipeline of simulation engines. These G-Buffers store rich data about materials, lighting, and geometry of virtual objects. Using the CARLA API, they transfer the intermediate G-Buffers from the simulation engine to the model environment. This approach aims to bridge the RG by improving the visual realism of simulation outputs, facilitating more reliable autonomous vehicle testing and research.

García-Daza et al. [12] analyzed the discrepancies in vehicle dynamics between simulated and real-world environments using a Model Predictive Controller (MPC) for trajectory planning. Their study employed statistical techniques—including MNCC to measure the similarity between simulation and real-world data. The evaluation of MPC performance focused on measuring the lateral error and control actions through tire angle measurements to evaluate the responsiveness and stability of the controller. For assessing comfort, the study analyzed lateral acceleration and jerk, to guarantee a comfortable ride experience for vehicle occupants. This research underscores the need for precise modeling of vehicle behavior to achieve seamless Sim2Real transitions, and the importance of addressing the RG in ADS development.

In addition, Hu et al. [13] conducted a comprehensive survey of methodologies to bridge the RG, categorizing them into three primary approaches: (i) transferring knowledge from simulation to reality, (ii) learning in digital twin, and (iii) learning by Parallel Intelligence (PI) technologies. Knowledge transfer or Sim2Real transfer techniques focus on training models in simulation and adapting them for real-world deployment. DT-based methods involve creating virtual replicas of physical systems, synchronizing real-time data to reflect lifecycle processes and enabling iterative improvements in simulation. PI emerges as an advanced approach that combines the strengths of Sim2Real and DT methods. PI creates an artificial system that mirrors the physical system, the ADS learns from both simulation and real-world data through computational experiments, and this knowledge is applied back to the physical vehicle in real-time using virtual–real interaction and online feedback, a process known as Parallel Execution (PE).

PE has emerged as a valuable tool for addressing RG by enabling synchronized interaction between real-world and virtual systems. In one of our previous works, Gutierrez-Moreno et al. [9] introduced our validation platform, which mirrors the movements of the real vehicle in its DT agent within the simulated environment using the PE method. This integration of the DT with the real vehicle enhances the fidelity and reliability of ADS validation by creating a feedback loop that aligns virtual and physical data streams.

Despite significant progress in addressing the RG, the concept of the Performance Gap (PG) has been less thoroughly explored [14,15]. Definitions from other fields, such as energy and building sciences, describe PG as the divergence between actual behavior and design intent [16]. Although this concept is less frequently addressed in the context of autonomous vehicles, it plays a crucial role in validation and the transition from simulated to real-world environments. In this domain, the PG is closely related to the RG and arises due to variations in dynamics, sensor behavior, processing times, latencies, and other factors, which can lead to inconsistencies in system performance when transitioning from simulation to the real world. During the early development phases, it is common to achieve high performance in simulations, only to observe a significant decline when moving to real-world environments—a challenge that is clearly attributable to the RG.

### Beyond the State of the Art: Our Proposal

The analysis of related works highlights significant progress in addressing the challenges of RG and, to a lesser extent, the PG in the context of AVs. Many studies focus on improving simulation fidelity or tackling specific aspects of ADS performance. For instance, Stocco et al. [10] propose using small-scale vehicles to bridge RG by focusing on lane-keeping controllers, while Pasios and Nikolaidis [11] focus on bridging the RG by enhancing simulation realism with photorealistic frameworks. However, these approaches often lack scalability, standardized metrics, and holistic methodologies to comprehensively address both RG and PG. In addition, the review of existing works highlights the lack of standardized evaluation metrics and ADS validation frameworks, while overlooking the need for holistic solutions that tackle the entire ADS. This highlights the importance of prioritizing system-level testing methods over model-level testing approaches.

Our approach addresses these limitations by proposing a unified methodology that integrates Digital Twin, Parallel Execution, and Real-World testing into an iterative framework. This approach simultaneously reduces RG and PG by holistically encompassing the entire ADS and systematically tuning simulator and ADS parameters, enabling consistent performance across environments.

In addition, we present an open-source Development and Validation Platform, designed to be affordable, reproducible, and accessible, enabling seamless transitions between simulation and real-world environments. By combining robust and holistic validation metrics with a structured transition from simulation to real-world environments, our methodology sets a foundation for more reliable ADS development. The results demonstrate its effectiveness in reducing key gaps, offering a scalable and reproducible framework for advancing research and practical applications in autonomous navigation.

## 3. Methodology for Closing Reality and Performance Gaps

Bridging the gap between simulation and real-world environments is a critical challenge in the development of ADS, which can be divided into two issues: the RG and the PG. The first refers to the discrepancies in behavior between a system tested in simulation and its behavior in real-world scenarios, primarily due to the inherent differences in environmental complexity, dynamics, sensory inputs, and HW dependencies. On the other hand, the PG highlights the difficulty of achieving an optimal parameter configuration that ensures consistent and robust performance across both domains, even when the simulation appears realistic.

Taken together, these issues amplify the challenge of aligning simulation behavior with real-world performance. Consequently, these discrepancies highlight the need for a systematic approach to ensure consistent performance and reliable validation across both environments.

**Figure 3:**
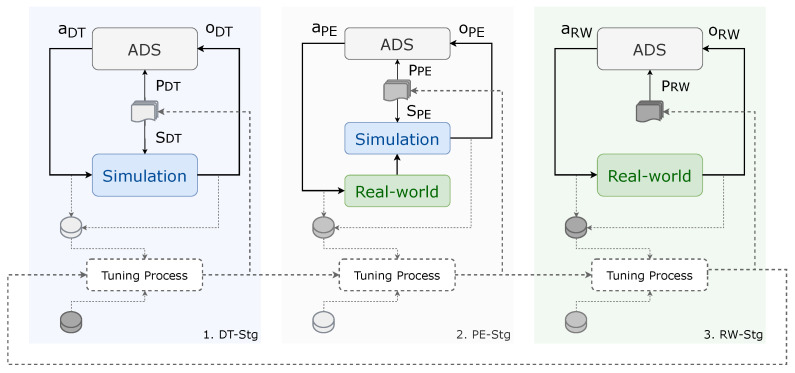
Methodology to close reality and performance gaps (MCRPG): Stages and data flow.

To address these challenges, we propose a structured methodology aimed at reducing both gaps, as illustrated in Figure 3. Our MCRPG consists of three complementary stages that progressively bridge the transition from simulation to real-world testing. These stages are as follows:

1. **Digital Twin Stage (DT-Stg)**: ADS is tested within a fully virtual environment without real-world interaction.
2. **Parallel Execution Stage (PE-Stg)**: the real vehicle and the DT agent in the simulator act as mirrors, performing the same movements in parallel. ADS generates actions that are applied to the real vehicle, which then transmits its real-time position to the agent in the simulator. The simulated environment generates the scenario’s objects and targets, producing observations that combine real-world dynamics with enriched virtual components for analysis and testing.
3. **Real-World Stage (RW-Stg)**: ADS operates fully in the real world.

A holistic comparison of the architecture’s performance across the three stages enables the identification and mitigation of gaps during the Sim2Real transfer process. This MCRPG introduces new possibilities that enhance development and validation processes beyond existing methods. It takes advantage of the strengths of simulation to provide a controlled and scalable environment for initial testing and parameter configuration, while systematically transitioning to real-world validation to ensure robustness and reliability.

Figure 3 illustrates the MCRPG approach proposed to bridge RG and PG. Before diving into the details of each stage, it is essential to define the key components, signals, and processes that constitute the core of the MCRPG:

- **Actions (*a*)**: represent any navigation outputs generated by the ADS. These outputs are generic and can include commands—such as linear and angular velocities, local trajectory, or other control signals—used for decision-making and navigation.
- **Observations (*o*)**: represent the data extracted from the environment through various sensors. This can include information such as positional data, jerk, steering angles, lateral errors, object detections, and other relevant metrics that provide situational awareness to the ADS.
- **ADS**: refers to the SW that is being developed, implemented, and enhanced. The proposed MCRPG is designed for a generic architecture capable of processing observations in real-time to generate corresponding actions.
- **Simulation**: The simulation encapsulates the ego-vehicle, sensors, test environment, targets, and the scenario generation tools. It is a hyper-realistic environment used to test and refine the ADS in a controlled and reproducible manner, allowing for extensive experimentation without the risks and costs associated with real-world testing.
- **Real-world**: The real-world environment includes the ego-vehicle, sensors, test environment, and targets. Unlike the simulation, this environment involves testing the ADS in live scenarios.
- **ADS’s Configuration Parameters (*P*)**: These parameters are intrinsic to the ADS and include settings like sampling rate, measurement errors, distance between waypoints, map name, max. steer angle, wheel track dist., sensor ranges, detection thresholds, and more. Tuning these parameters allows for minimizing the PG by improving system performance.
- **Simulator’s Setting Parameters (*S*)**: These parameters govern the simulation environment and include factors such as synchronous mode, frame rate, map name, ego mass, torque curves, sensor ranges, sensor noises, weather conditions, agent dynamics, and more. Tuning these parameters can reduce the RG by making the simulation more representative of real-world conditions.
- **Tuning Process**: In this work, the tuning process employs a derivative-free coordinate-search optimization over predefined ranges of *P* and *S*. For each parameter, a small discrete set of candidate values is evaluated using the logs of actions and observations, and the value that maximizes the reality-alignment and ego-performance metrics. This procedure is repeated iteratively until no further improvement is observed.

As shown in Figure 3, the three phases form a feedback loop where logs of actions (*a*) and observations (*o*) are stored and analyzed after simulation for iterative optimization within the Tuning Process, with two main goals: optimize *P* to improve the performance bridging PG; and optimize *S* to reduce the RG, ensuring consistent behavior in both simulation and real-world scenarios.

By iteratively refining both *P* and *S* parameters through a feedback loop involving calibration and fine-tuning, our method aims to achieve behavioral consistency between simulated and real-world environments, while maximizing the performance of the ADS in real-world conditions. The following subsections break down each phase in detail, discussing their implementation, and contributions to closing the gaps:

### 3.1. Digital Twin Stage

In this first stage, the ADS interacts with the simulator-generating control actions ($a_{DT}$) that are fed into the simulated vehicle and environment. Observations ($o_{DT}$) from the simulator, based on its setting parameters, are returned to the ADS in an online loop:

(1) $$a_{DT}=f_{ADS}\left(o_{DT},P_{DT}\right)$$

(2) $$o_{DT,t+1}=g_{Sim}\left(a_{DT},S_{DT}\right)$$

where:

- $P_{DT}$: ADS parameters (tunable).
- $S_{DT}$: Simulator settings (tunable).
- $f_{ADS}$: ADS control function governed by $P_{DT}$.
- $g_{Sim}$: Simulator model governed by $S_{DT}$.

Initially, there are no preconfigured values for $P_{DT}$ and $S_{DT}$, so default parameters are used, which may not perform optimally. The goal is to refine these parameters iteratively by repeating the loop multiple times. The interaction between ADS and the simulator generates a data log which is stored for offline fine-tuning:

(3) $$D_{DT}={\left\{\left(a_{DT}^{\left(i\right)},o_{DT}^{\left(i\right)}\right)\right\}}_{i=1}^{N}$$

The block referred to as Tuning Process in Figure 3 represents a procedure where we analyze the current data logs, $D_{DT}$, along with previously recorded data logs, $D_{RW}$, to perform fine-tuning both $P_{DT}$ and $S_{DT}$. This process is based on pre-tuning values derived from RW-Stg. The optimization equation for tuning $S_{DT}$ parameters is defined as follows, aiming to reduce the current RG. In other words, the goal is to minimize the difference between the current observations and the real-world observations obtained during the previous stage:

(4) $$S_{DT}^{*}=\arg\min_{S_{DT}}{∥o_{DT}-o_{previous\_RW}∥}^{2}$$

An example of tuning could be the following: If DT-Stg reports a speed for the ego-vehicle of 8 m/s and previous RW-Stg reports 7.5 m/s, we adjust the ego-vehicle’s physics by modifying relevant simulator settings in $S_{DT}$. One such setting is the tire friction coefficient, which affects the ego’s interaction with the road. By refining this parameter in the simulator, we ensure that the simulated dynamics better align with real-world behavior. The Tuning Process then updates the ADS parameters to optimize its performance ($J_{ADS}$), ensuring that the ADS achieves the desired behavior under optimized settings:

(5) $$P_{DT}^{*}=\arg\max_{P_{DT}}J_{ADS}\left(o_{DT},a_{DT}\right)$$

Another example of tuning for $P_{DT}$ could involve adjusting the parameters of the ADS’s Tracking Controller, such as the weights in an MPC cost function. Suppose that, in the simulation, the ego-vehicle exhibits excessive lateral deviations when following a reference trajectory, leading to unstable behavior. To address this issue, we can optimize $P_{DT}$ by modifying the weight assigned to lateral error in the cost function, prioritizing trajectory accuracy over control effort. By fine-tuning this parameter, the ADS achieves a more stable and precise tracking performance, better aligning with real-world driving behavior and improving its generalization to other environments.

This stage establishes the foundation for fine-tuning both the simulator settings and the ADS behavior. In practice, the optimization problems in Equations (4) and (5) are solved using the above coordinate-search routine: in each iteration, we sweep a small grid of candidate values for one parameter while keeping the remaining parameters fixed, and we keep the configuration that yields the best behavior, meaning the minimum difference between observations and maximum performance. The optimized parameters are then validated and refined in the next phase, ensuring a smooth transition toward real-world execution.

### 3.2. Parallel Execution Stage

The PE-Stg bridges the gap between simulated behavior (DT-Stg) and real-world behavior (RW-Stg) by combining simulated observations with real-world execution. This approach allows the system to refine its parameters using real-time feedback while maintaining alignment with the simulation, incorporating real vehicle dynamics to enhance realism. In this stage, observations ($o_{PE}$) are generated by the simulator, and actions ($a_{PE}$) are sent to the real vehicle:

(6) $$a_{PE}=f_{ADS}\left(o_{PE},P_{PE}\right)$$

(7) $$o_{PE,t+1}=g_{Sim}\left(a_{PE},S_{PE}\right)$$

The discrepancy between $o_{PE}$ (observations from PE-Stg) and $o_{DT}$ (from DT-Stg) provides insights into how accurately the simulator reflects real-world behavior. Minimizing this difference is critical for improving simulation fidelity:

(8) $$S_{PE}^{*}=\arg\min_{S_{PE}}{∥o_{PE}-o_{DT}∥}^{2}$$

Additionally, we refine the optimization of $P_{PE}$ to ensure improved system performance using the real vehicle. The optimization equation for tuning $P_{PE}$ parameters is defined as follows:

(9) $$P_{PE}^{*}=\arg\max_{P_{PE}}J_{ADS}\left(o_{PE},a_{PE}\right)$$

The results from PE-Stg provide optimized parameters and improved simulation accuracy using the same coordinate-search procedure, preparing the system for real-world testing and validation in the next RW-Stg stage. This approach also facilitates continuous interaction between simulated and real environments, ensuring that discrepancies are progressively reduced. By integrating the simulator and real vehicle in a feedback loop, this stage promotes the convergence of RG and PG, enabling a seamless transition to real-world scenarios.

### 3.3. Real-World Stage

RW-Stg represents the last step in the validation loop, where the ADS is tested in real-world conditions. After parameter refinement through DT-Stg and PE-Stg steps, this stage focuses on evaluating the performance in the real-world scenario, addressing any remaining discrepancies or improvements. In this stage, the ADS operates entirely in the real-world environment, with actions and observations derived directly from physical interactions:

(10) $$a_{RW}=f_{ADS}\left(o_{RW},P_{RW}\right),\;o_{RW,t+1}=g_{real}\left(a_{RW}\right)$$

Unlike previous stages, no simulator is involved in RW-Stg. Instead, the Tuning Process focuses on fine-tuning the ADS’s $P_{RW}$ parameters by using the explained coordinate-search method, based on real-world logs, to achieve optimal system performance ($J_{ADS}$):

(11) $$P_{RW}^{*}=\arg\max_{P_{RW}}J_{ADS}\left(o_{RW},a_{RW}\right)$$

This stage is critical for validating the robustness and reliability of the ADS in the driving scenario, where the complexity of real-world environments challenges the system’s adaptability. This process ensures that the ADS can effectively handle diverse conditions, such as environmental factors, limited computational resources, latency in data transmission, synchronization issues between distributed modules, sensor noises, and other challenges which may not be fully replicated in simulation.

### 3.4. Iterative Cycle

The RW-Stg completes the cycle by feeding $P_{RW}$ and $S_{RW}$ = $S_{PE}$ parameters back into the DT-Stg, ensuring continuous alignment between simulation and reality.

The success of this iterative cycle is primarily evaluated by the progressive reduction of the three key gaps: the Reality Gap (RG), the Performance Gap in simulation ($PG_{sim}$), and the Performance Gap in real-world ($PG_{real}$). The main objective of this cycle is to drive these gaps toward zero:

(12) $$\lim_{t\to \infty }\mathrm{RG}=0,\;\lim_{t\to \infty }{\mathrm{PG}}_{sim}=0,\;\lim_{t\to \infty }{\mathrm{PG}}_{real}=0$$

Achieving this objective is highly challenging and depends on multiple factors, including the level(s) at which behavior is analyzed, the complexity of both the system and the environment, how realistic the simulator is, the agents’ models, and more. Therefore, these equations describe an ideal state that we attempt to reach iteration by iteration. Convergence toward this ideal is essential to ensure a seamless and reliable transfer of the ADS capabilities from the simulated environment to the real world, without compromising the system’s performance in either domain.

To address this challenge, the iterative cycle focuses on progressively reducing the discrepancies between the actions executed by the system in the simulator and those performed in the real world, as well as the difference between observations in both environments. These can be expressed mathematically as:

(13) $$\lim_{t\to \infty }∥a_{DT}-a_{RW}∥=0,\;\lim_{t\to \infty }∥o_{DT}-o_{RW}∥=0$$

The reduction of discrepancies in both actions and observations is what allows the RG, $PG_{sim}$, and $PG_{real}$ to converge toward zero, demonstrating that the system has achieved perfect alignment between the two environments and optimized performance. This iterative process continues until the differences converge, ensuring that the system is robust and consistent both in simulation and in real-world scenarios.

## 4. Our Development and Validation Platform (DVP)

Building a robust ADS requires a holistic approach that connects simulation with real-world validation. We introduce a versatile and robust DVP which integrates those three key components: the ADS, the simulation framework, and the real-world environment.

This SW and HW platform for developing and refining the ADS was proposed by the authors in [17]. Our ADS core concept remains unchanged: a powerful ROS-based [18] modular architecture made up of several state-of-the-art reference implementations for the different layers of autonomous navigation.

This DVP facilitates the Sim2Real transfer of our ADS and allows us to analyze and reduce the current RG and PG by comparing the performance of our stack in the three stages. The first stage consists of using the DT approach, where we perform several urban scenarios within our virtual university campus. This stage involves creating a custom map for CARLA, including road layout, HD map, static and dynamic objects, sensors, and vehicles. The second phase consists of performing the same scenarios following the PI validation approach, where interaction with adversarial vehicles is simulated using virtual perception through a parallel execution, while the ADS is evaluated using our real vehicle. Finally, the third stage involves executing the scenarios in a fully real-world environment, using our ego-vehicle and adversarial elements within our campus.

The idea emerged from the need to implement and validate a modular architecture based on ROS. This open-source framework provides powerful tools for node management, communication, synchronization, and integration of diverse libraries, offering flexibility and efficiency in ADS development. Using ROS allows the platform to run on any GPU-powered device, ensuring scalability and accessibility.

**Figure 4:**
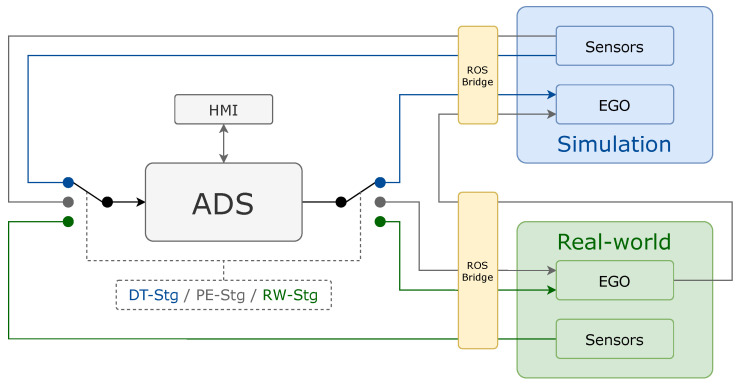
Development and validation platform (DVP) overview: Bridging digital twin and real-world stages via parallel execution.

As we show in Figure 4, one of the key strengths of this platform lies in its ability to seamlessly switch between DT-Stg, PE-Stg and RW-Stg testing. Through the use of simulators and the integration of a ROS bridge interface, the ADS can interact with a virtual environment. Similarly, when working with an ego-vehicle in a real-world environment, the same architecture can process inputs from physical perception sensors (e.g., GNSS, LiDAR and cameras) compatible with ROS.

In all cases, the ADS module serves as the main component that processes observations and measurements from the sensors and generates control commands for the ego-vehicle. This flexibility simplifies transitions between virtual and physical testing, allowing developers to validate algorithms in simulation before deploying them in real-world environments.

The modular nature of the platform ensures that individual components, such as perception, control, or planning modules, can be independently updated, tested, and validated. This modularity is particularly advantageous when integrating new sensors, upgrading SW, or extending the system’s capabilities. Additionally, thanks to ROS’s communication framework, the platform supports real-time synchronization and smooth data flow between the ADS and its external environment, whether simulated or real.

Switching between simulation and real-world execution has been traditionally challenging due to the differences in communication protocols, HW setups, and data formats. The proposed platform solves this problem by creating a unified interface that supports both environments. Developers can launch and test the ADS in simulation to fine-tune performance, and then deploy the same ADS configuration on an ego-vehicle without additional modifications. This approach reduces development time, enhances reliability, and enables continuous iteration.

In conclusion, this work presents a robust, open-source, cost-effective, and flexible DVP capable of implementing and validating ADS in both simulated (SW) and real-world (HW) environments.

Regarding the SW, our platform is based on CARLA [19], a hyper-realistic open-source simulator developed to design and validate autonomous driving systems. This simulator provides us with tools to define agent dynamics, create scenarios, vary weather conditions, establish different sensor distributions, generate maps, and much more.

As for the HW, we have implemented an open-source autonomous electric car, which is affordable and easily reproducible by third parties. We decided to build our own autonomous vehicle from scratch, covering all the design phases involved in this process: mechanics, electronics, localization, perception, planning, control, and navigation. This platform provides us with all the necessary HW tools to validate an autonomous architecture in a real-world environment.

In the following subsections, we provide an overview of the three key components that make up this platform: the ADS, the simulation framework and the real-world platform.

### 4.1. ADS: An Overview of Our Autonomous Driving Stack

While a detailed analysis of the ADS is beyond the scope of this paper, we include a brief overview of the architecture used throughout our experiments to contextualize the proposed methodology. Our main objective is not to evaluate or improve the ADS itself, but rather to propose and validate a methodology for minimizing RG and PG using a general-purpose ADS, that means any autonomous driving SW capable of generating actions based on real-time observations.

For an in-depth technical description of our ADS, we refer the reader to our previous work [17]. That work presents our ADS: built from scratch, modular, open-source architecture developed using ROS and Docker. ROS enables communication and coordination across distributed modules through a publish/subscribe messaging mechanism over Inter-Process Communication (IPC), while Docker provides lightweight containerization of each functional layer, ensuring reproducibility, portability, and simplified deployment.

**Figure 5:**
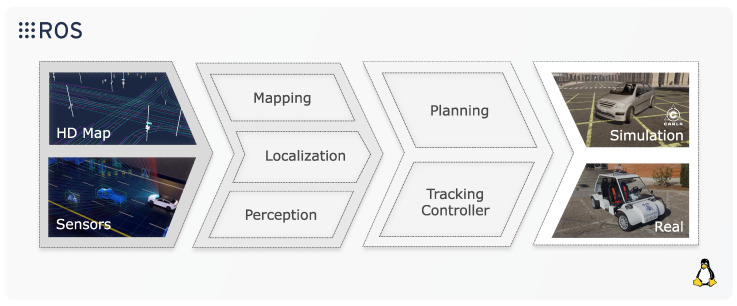
Our Autonomous Driving Stack (ADS).

As illustrated in Figure 5, our ADS follows a layered, hierarchical, and decoupled design, which enhances maintainability, reusability, and scalability. Each layer can be independently replaced, upgraded, or validated, facilitating systematic development and testing. The main layers of the architecture are:

- **Mapping**: This layer is built upon OpenDRIVE, a standardized HD Map format developed by Association for Standardization of Automation and Measuring Systems (ASAM) to represent static road networks for driving simulation environments. As detailed in [20], it is divided into two main modules:
  – Map Parser: Processes the HD Map and extracts relevant information like lane geometries and traffic light positions.
  – Map Monitor: Continuously monitors the vehicle’s surroundings using its current position, map data, and planned local trajectory. It identifies relevant lanes and regulatory elements, such as the current lane, upcoming intersection lanes, crosswalk areas, traffic lights, and more.
- **Localization**: Combines Global Navigation Satellite System (GNSS) and wheel odometry data through an Extended Kalman Filter (EKF) to estimate the vehicle’s pose in real-time. It also publishes the transform tree that links all coordinate frames (e.g., world, map, ego-vehicle, sensors or other attached elements). This transform system enables ROS to compute frame transformations efficiently, one of the key strengths of the middleware.
- **Perception**: Implements sensor fusion techniques using LiDAR and camera data.
  – Camera: Uses YOLOv8 [21] for real-time object detection in the images. Bounding boxes are projected into 3D using the camera’s intrinsic parameters to approximate object location.
  – LiDAR: Employs the MMDetection3D [22] framework with the PointPillars architecture for 3D object detection. The model detects and clusters dynamic objects, such as vehicles and pedestrians.
  – Object Fusion: Combines detections from both sensors using a late fusion approach to ensure accurate data association and to integrate complementary detection information. The fusion module outputs an Oriented Bounding Box (OBB) for every object, along with its 2D pose (x, y, yaw) relative to the ego-vehicle.
- **Planning**: This layer is divided into two main modules:
  – Global Path Planning: Computes the optimal global route using a Dijkstra-based graph algorithm, generating a topological path composed of roads and lanes. It includes a Lane Graph Planner (LGP), which builds the route graph by considering lane connections and associated travel costs, and a Lane Waypoint Planner (LWP), which discretizes the path into 3D waypoints spaced at fixed intervals for navigation. Additional details can be found in [20].
  – Decision-Making: Based on Hierarchical Interpreted Binary Petri Nets (HIBPNs), this module encodes discrete driving behaviors such as traffic light handling, pedestrian crossings, emergency stops, and other rule-based traffic interactions. Refer to [23] for further details.
- **Tracking Controller**: This module follows a sequence of waypoints by generating a smooth trajectory using cubic spline interpolation. Lateral control is handled through a Linear Quadratic Regulator (LQR), which computes the steering angle command sent to the Steer-By-Wire (SBW). Longitudinal control is performed independently, the desired speed is dynamically adjusted based on path curvature through a predefined speed profile, and the Throttle-By-Wire (TBW) regulates the throttle/brake commands to achieve the requested speed. In addition, a delay compensation module predicts future positions to mitigate the impact of actuator delays. Furthermore, the tracking controller works jointly with an MPC, which introduces a smooth offset in the trajectory when obstacle avoidance is required, ensuring safe maneuvering without abrupt deviations. This approach ensures accurate and stable tracking performance, especially in urban driving scenarios. For an in-depth explanation, refer to [24].

This modular design not only enables seamless integration with both simulation and real-world environments but also simplifies debugging, validation, and performance tuning across the different stages of our methodology.

### 4.2. Simulation: CARLA as Our Framework

To simulate and evaluate our methodology within a realistic and adaptable environment, we utilize CARLA, a high-fidelity, open-source simulator specifically developed for autonomous driving research. CARLA provides a comprehensive suite of tools for defining agent dynamics, environment conditions, sensor setups, and scenario generation. These capabilities make it an ideal platform for modeling complex urban environments and validating AV behaviors under a wide range of conditions [19].

To integrate CARLA with our ROS-based architecture, we leverage the CARLA Python Application Programming Interface (API). This API enables the implementation of custom ROS bridge nodes that facilitate interaction with the ego-vehicle, agent creation, script execution, sensor placement and readings, as well as bidirectional communication between CARLA and the ADS. The bridge is responsible for reading sensor data and vehicle states from the simulator, while also sending the generated control commands back to the simulator in real-time. This setup ensures seamless interaction and tight synchronization between the simulation and the SW stack.

**Figure 6:**
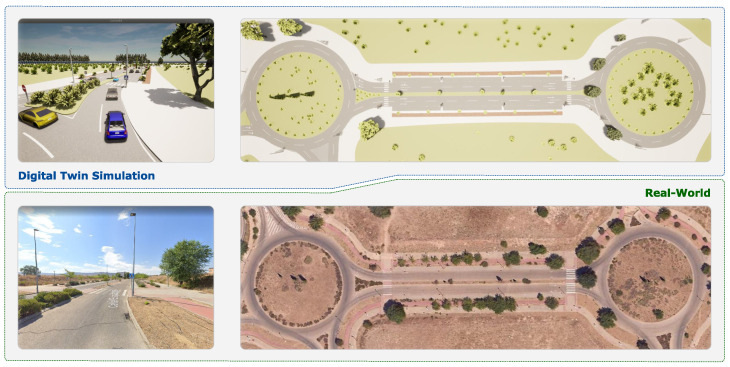
Validation environments: Digital twin vs. real-world.

As part of our simulation framework—and following the official CARLA tutorial for map creation [25]—we manually created a custom HD Map representing our university campus. This map was developed using RoadRunner-VectorZero [26], which provides comprehensive road geometry, detailed layouts, traffic elements, and other environmental features, enabling highly realistic scenario validation. Once the map was completed, we exported it to Unreal Engine, generating two essential files: the road network file in OpenDRIVE format (.xodr), and a 3D model file (.fbx) containing detailed environmental information. Unreal Engine, responsible for physics simulation and rendering, also provides flexibility for environment customization. Figure 6 illustrates the proving ground generated from this process, which we used during our experiments.

Furthermore, we designed and implemented urban driving scenarios—using CARLA’s ScenarioRunner tool [27]. This tool enables the creation and execution of complex traffic scenarios within the simulator. Scenarios can be defined through a Python API or using the ASAM OpenSCENARIO standard. For the purpose of our validation, we employed OpenSCENARIO, a standard that focuses on modeling the temporal and behavioral interactions of traffic participants—such as vehicles, pedestrians, and other agents—alongside environmental conditions like weather and time of day [28]. These scenarios involve dynamic elements, including the movements of pedestrians and vehicles, enabling the evaluation of critical behaviors such as pedestrian crossing, adaptive cruise control, and more. This setup allows the ADS to be tested under diverse and challenging conditions, facilitating performance analysis and system parameter tuning.

Overall, CARLA serves as a fundamental element of our simulation framework, providing a reproducible and configurable environment designed for iterative testing and validation. Its flexibility supports the comprehensive development lifecycle of autonomous driving systems, bridging the gap between preliminary simulation-based evaluations and deployment in real-world scenarios.

### 4.3. Real-World: TABBY EVO as Our HW Platform

Due to the high costs of commercial solutions for having an automated vehicle—and the fact that these solutions are proprietary and closed systems—we opted to build our own HW platform from scratch based on an open-source chassis. For this, we selected the TABBY EVO platform developed by Open Motors [29].

The chassis was originally equipped with a 19-kW asynchronous AC motor, an AC-L1 controller from the Italian group SME, a BMS SCC24 from the XBW brand and a ZIVAN NG3 charger that sends 80 V and 25 A to the batteries. The technical characteristics of the vehicle are shown in Table 1.

**Figure 7:**
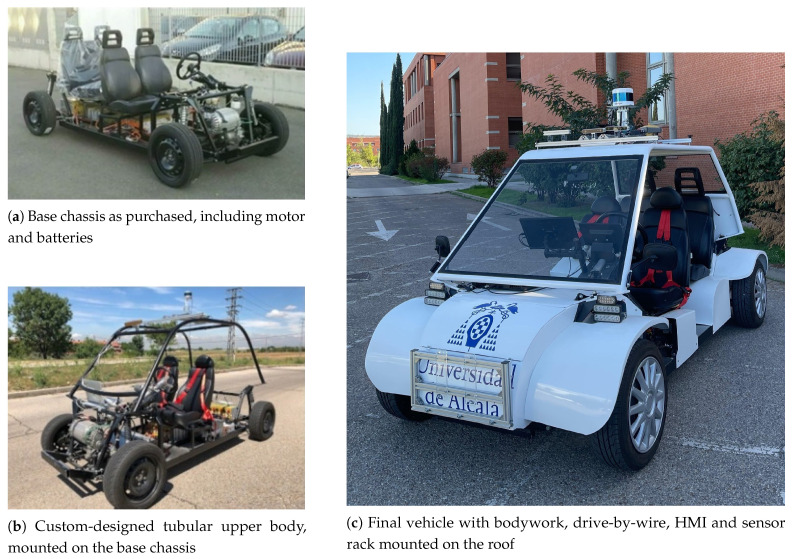
TABBY EVO development: From initial chassis to final HW platform.

In the first stage of development, we installed the battery pack and designed a tubular steel chassis. Subsequently, a steel bodywork was built and mounted over this chassis. An aluminum roof rack was added to house all perception and localization sensors, along with the necessary HW for autonomous navigation. The construction phases of the vehicle are illustrated in Figure 7.

To enable autonomous control, we developed a Drive-By-Wire (DBW) system entirely from scratch. This allows control of linear velocity and steering angle through commands sent via a local network from the main Edge Computing Unit (ECU). These commands are processed by a dedicated control board running FreeRTOS for real-time performance. Two independent anti-windup PID controllers were implemented—one for each controlled variable.

The SBW was first implemented by replacing the mechanical steering column with an electric one and designing a custom angular position sensor to provide feedback to the PID controller. With a very small $K_{i}$ constant, this classical approach is highly suitable and well-proven for steering applications. Due to the hardware limitations, it is widely used in commercial vehicles, autonomous driving and trajectory tracking research [30]. Additionally, the LQR lateral controller is running at the high level for providing robustness against control disturbances.

Subsequently, we developed the TBW module using a Digital-to-Analog Converter (DAC) to generate the appropriate throttle signal for the traction motor controller. An encoder on the motor provides the necessary feedback for the control loop. A Brake-By-Wire (BBW) was not included, as the regenerative braking system met the vehicle’s requirements. Additionally, a manual/automatic switching system allows the driver to safely activate or deactivate autonomous mode at any time. A ROS node was implemented on a Raspberry Pi to receive velocity and steering commands and translate them into the controllers. Full technical details of the DBW are provided in [31].

The vehicle’s pose estimation system fuses GNSS data with encoder-based odometry. To enhance accuracy and reliability, we employ Differential GNSS (DGNSS) and Real-Time Kinematic (RTK) positioning techniques [32]. The system uses a Topcon MR-2 receiver capable of tracking all satellite constellations, such as GPS, GLONASS, Galileo, and BeiDou. Odometry estimates the vehicle’s motion using two Kübler 8.3700.1322.0360 optical incremental encoders mounted on the rear wheels. While odometry accumulates errors over time, it is low-cost, simple, and effective in short periods, especially when GNSS signals degrade. The robot_localization package provided by ROS merges both data using an EKF, enabling real-time, centimeter-level accuracy [33].

The perception system combines data from a Velodyne Alpha Prime VLS-128 LiDAR and a StereoLabs ZED X RGB camera, both mounted on the roof rack. This setup allows the detection and classification of both static (e.g., traffic signs, road markings) and dynamic (e.g., vehicles, pedestrians, cyclists) objects in the environment.

Given the high computational demands and real-time processing requirements of autonomous driving, the system is distributed across six dedicated ECUs:

- **Localization (RearECU)**: Raspberry Pi 4 Model B (located in the rear trunk).
- **Drive-By-Wire (DBW)**: Raspberry Pi 4 Model B (installed near the steering wheel).
- **Human–Machine Interface (HMI)**: Jetson Xavier NX (mounted near the dashboard screens).
- **Perception (RackECU)**: Jetson AGX Orin (mounted near the camera and LiDAR on the roof).
- **Main PC**: HP Omen 17-CK0000/Intel i7-11800H/32GB RAM/RTX 3070. Responsible for executing all remaining processes not handled by other units, in addition to launching and orchestrating all distributed processes.
- **Secondary PC**: Identical to the main PC, used for running CARLA during the PE-Stg.

**Figure 8:**
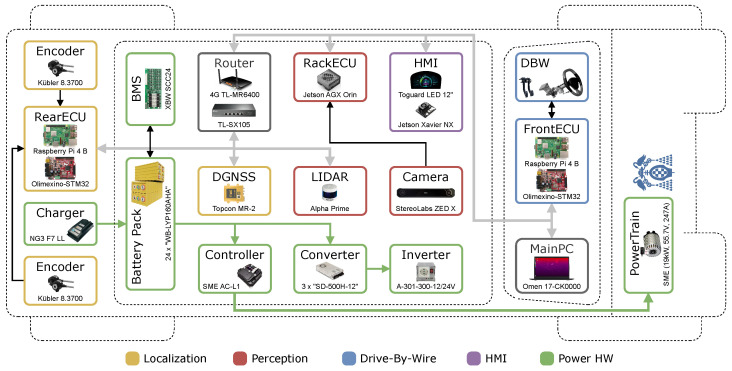
TABBY EVO hardware architecture.

The overall HW architecture of the TABBY EVO, including sensor and device placement, as well as interconnections, is illustrated in Figure 8. This platform not only serves as a complete physical testbed for real-world validation of our ADS, but is also open-source, modular, and reproducible, allowing other research teams to replicate and extend it for their own autonomous navigation experiments.

## 5. Experiments

In this section, the experiments conducted to evaluate the proposed MCRPG methodology for closing the RG and the PG gaps during the development of an ADS are described. The experiments are structured into three key stages: DT-Stg, PE-Stg and RW-Stg. These stages not only enable the evaluation of the ADS performance in a controlled and dynamic environment but also facilitate an iterative analysis to optimize the configuration parameters of both the ADS and the simulator.

The following subsections detail the design of the test scenario (Section 5.1), the evaluation metrics applied (Section 5.2), and the results obtained across the different stages (Section 5.3).

### 5.1. The Scenario: Design and Generation

Before proceeding with the experimental results, it is crucial to highlight the importance of designing and generating testing scenarios. This process is essential for the development of AVs, as the richness and relevance of scenarios directly impact the effectiveness of validation processes. Scenario design is crucial for exposing ADS to diverse challenges and achieving reliable performance.

Our MCRPG method inherently includes the creation of scenarios with sufficient diversity and complexity to validate the architecture across varied use cases. Richer scenarios significantly contribute to reducing both the RG and PG, enhancing the transferability of simulation findings to real-world conditions. However, this richness also introduces additional complexity to the validation process, requiring meticulous data analysis and parameter fine-tuning.

Extensive research has focused on addressing the challenges of scenario generation. As highlighted in [34], Scenario-Based Testing (SBT) is one of the most widely adopted approaches for verification and validation in AV research, with scenarios being the central artifact of this process. The authors in [35] classify SBT scenarios into categories such as critical, challenging, complex, and more. Critical scenarios, for instance, are those where the ego-vehicle leads to or narrowly leads to collisions, often characterized by metrics like Time To Collision (TTC). These scenarios test the limits of the ADS’s decision-making capabilities, ensuring safety under high-risk conditions. Reference [36] proposes the concept of Critical Boundary Driving Scenarios (CBDS), defined as naturalistic yet safety-critical situations that expose the performance limits of AVs.

**Figure 9:**
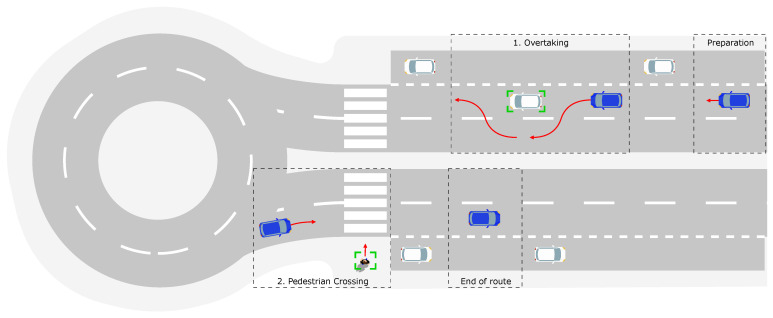
The scenario: Urban driving environment with concatenated use cases.

Following this framework, our work adopts the SBT approach to design a complex and realistic validation environment. We focus on an urban driving scenario designed to evaluate the performance of the ADS. The scenario involves low-speed urban driving, with a maximum legal speed limit of 30 km/h, over a total route length of approximately 300 m—as illustrated in Figure 9.

The ego-vehicle begins in a preparation zone and autonomously follows the route, executing a lane keeping behavior until it reaches the final destination specified by the driver. Along the way, the ego-vehicle must safely handle two sequential use cases:

1. **Overtaking**: While traveling at a speed below 30 km/h, the ego-vehicle encounters a stationary target vehicle in its lane. To proceed, the ego-vehicle must detect the obstacle and perform a safe overtaking maneuver.
2. **Pedestrian Crossing**: Continuing at a speed below 30 km/h, the ego-vehicle approaches a pedestrian crossing. Here, it must detect and respond appropriately to a pedestrian crossing the street at a speed of approximately 2.0 m/s, moving perpendicularly across the ego-vehicle’s path.

This combined scenario is defined as a CBDS, since it involves two sequential and safety-relevant events that test both the longitudinal and lateral dynamics of the vehicle. The scenario is defined following the OpenSCENARIO standard [28], ensuring reproducibility within simulation platforms.

### 5.2. The Evaluation Metrics

The Tuning Process, introduced in Section 3, is implemented as a coordinate-search optimizer that performs iterative adjustments of parameters based on logs that record actions and observations from each stage. By fine-tuning these parameters, discrepancies between simulated and real-world environments can be systematically minimized, effectively reducing both the RG and the PG. The more exhaustive the analysis, the greater the extraction of valuable information from the scenario and from the ADS performance, enabling precise parameter adjustments and enhancing system reliability. However, more thorough analysis introduces additional challenges, particularly in tasks such as data integration, ensuring data quality, and managing the complexity of large datasets.

However, developing reliable evaluation frameworks for AVs remains a critical challenge. No unified standard currently exists for assessing ADS performance due to the complexity and multidimensionality of driving behavior. Evaluating AVs objectively in both simulated and real-world environments remains a challenge. Numerous studies highlight the importance of identifying effective evaluation metrics, performance indicators, and key variables, all with the goal of establishing a more consistent and standardized evaluation process.

For instance, the authors in [37] proposed a set of Behavior Metrics based on CARLA’s Leaderboard Driving Score, providing a more informative and complementary perspective on ADS behavior. They included metrics such as Mean Position Deviation per Km, Vehicle Longitudinal Jerk per Km, GPU Inference Frequency, among others. The study in [38] introduced a comprehensive AV evaluation framework based on four aspects: safety, comfort, driving performance, and standard regulations. This work is much more extensive and also proposes principles for the design of test scenarios.

Building on this foundation, the authors in [39] expanded this framework by proposing the Objective Multi-Dimensional Comprehensive Evaluation (OMDCE) method that divides the intelligent driving test into four modules: test scenarios, scenario complexity model, simulation test platform, and automated evaluation system. The scenario complexity model is proposed to bridge the gap between the test scenarios and the evaluation system, enabling adaptive scaling of the evaluation scale with different difficulty levels. Additionally, OMDCE adds a fifth aspect, called Traffic Coordination, to the four Ego Performance aspects already defined, positioning it as an Altruism performance aspect.

**Figure 10:**
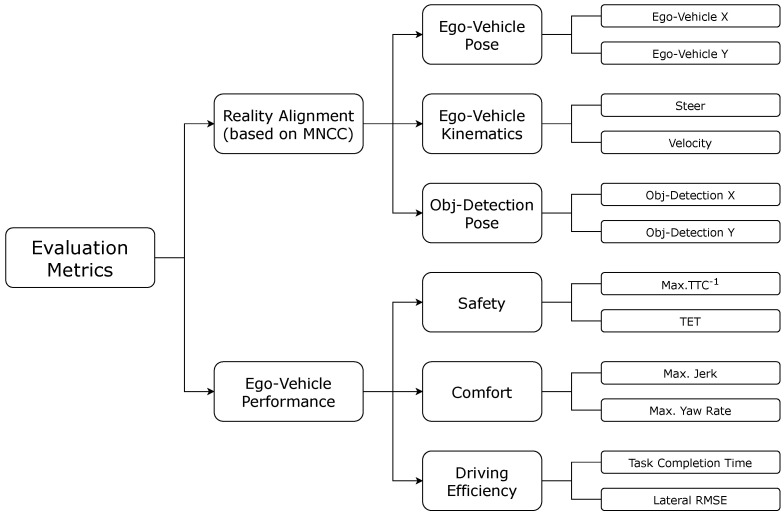
Evaluation metrics.

In line with previous approaches, our work adopts a structured evaluation framework that integrates multiple grouped metrics, as illustrated in Figure 10. These metrics are organized into two main categories: Reality Alignment Metrics—based on MNCC—and Ego-Vehicle Performance Metrics.

The first category encompasses measures related to the alignment between simulated and real-world driving behavior, including metrics for Ego-Vehicle Pose, Ego-Vehicle Kinematics, and Object-Detection Pose. The second category evaluates the performance aspects of the ego-vehicle through indicators of Safety, Comfort and Driving Efficiency. Collectively, these metrics enable a comprehensive quantification of ADS behavior across the three experimental stages: DT-Stg, PE-Stg, and RW-Stg.

#### 5.2.1. Reality Alignment Metrics

The Reality Alignment metrics evaluate the fidelity of the simulation relative to real-world behavior by comparing signals from the current stage with those from the previous one. In MCRPG, the comparisons are performed sequentially, starting with ***DT-Stg* vs. *previous_RW-Stg***, followed by ***PE-Stg* vs. *DT-Stg***, and finalizing with ***RW-Stg* vs. *PE-Stg***—completing a continuous evaluation cycle. This analysis relies on the MNCC method to assess signal similarity, in the same way as the authors of [12]. For each signal *x* (e.g., ego-vehicle position, steering-wheel angle, velocity, position, etc.) between the current stage ($x_{\mathrm{current}}$) and the previous stage ($x_{\mathrm{previous}}$), MNCC is defined as:

(14) $$\mathrm{MNCC}\left(x\right)=\frac{\sum_{i}\left(x_{\mathrm{current}}\left(i\right)-\mu_{\mathrm{current}}\right)\left(x_{\mathrm{previous}}\left(i\right)-\mu_{\mathrm{previous}}\right)}{\sqrt{\sum_{i}{\left(x_{\mathrm{current}}\left(i\right)-\mu_{\mathrm{current}}\right)}^{2}\sum_{i}{\left(x_{\mathrm{previous}}\left(i\right)-\mu_{\mathrm{previous}}\right)}^{2}}}$$

where:

- $\mu_{\mathrm{current}}$ is the mean value of $x_{\mathrm{current}}$.
- $\mu_{\mathrm{previous}}$ is the mean value of $x_{\mathrm{previous}}$.

The resulting MNCC value ranges from −1 to 1, where 1 denotes perfect alignment between signals from consecutive stages. MNCC evaluates temporal offsets between signals; therefore, all signals are recorded from a common trigger event (e.g., route start or ADS activation). This ensures that signals from different runs share the same temporal reference point.

To address variations in signal duration between runs, we apply a temporal cropping procedure so that both $x_{\mathrm{current}}$ and $x_{\mathrm{previous}}$ have the same time length before computing MNCC. This prevents distortions caused by mismatched time spans and ensures that the metric compares equivalent temporal segments.

By combining synchronized start times with equalized signal lengths, the resulting MNCC values provide a more valid and robust measure of similarity between simulation and real-world signals. In our case, we decided to analyze six representative signals: the vehicle’s 2D pose (Ego-Vehicle X, Ego-Vehicle Y), steering angle (Steer), linear velocity (Velocity), and the 2D pose (Obj-Detection X, Obj-Detection Y) of the target object that the Decision-Making module identifies as the highest-priority element in the scene.

#### 5.2.2. Ego-Vehicle Performance Metrics

To evaluate the performance of the ego-vehicle, we define a set of metrics grouped into three key categories: *safety*, *comfort*, and *driving efficiency*. These metrics allow for a quantitative assessment of the ADS behavior throughout the different stages and scenarios of the proposed MCRPG methodology (DT-Stg, PE-Stg, RW-Stg), providing a solid foundation for parameter tuning and comparison between simulated and real-world domains.

(a) Safety Metrics

These metrics assess the ADS’s ability to avoid hazardous situations or potential collisions during autonomous navigation.

**Figure 11:**
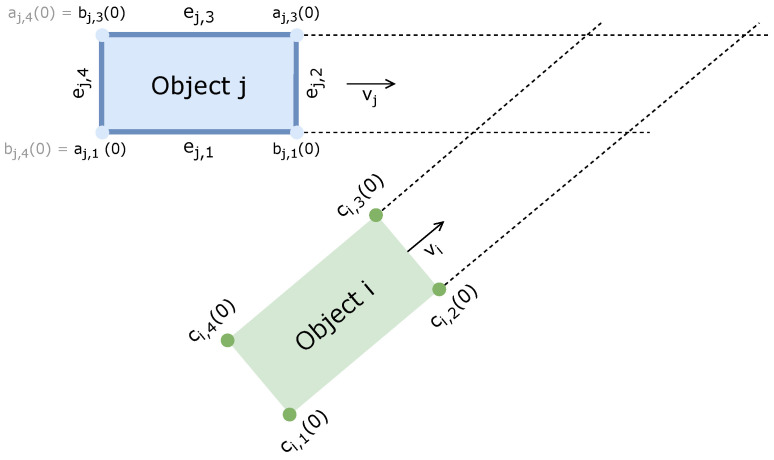
TTC approach: intersection analysis between two moving OBBs.

- **Maximum Inverse Time-To-Collision (Max. TTC^−1^):** We employ a geometric TTC estimator based on the corner–edge collision method introduced in [40], which enables the handling of oriented objects moving along arbitrary 2D trajectories by representing each object with OBBs, as we show in Figure 11. Under constant velocity and fixed orientation, the TTC is obtained by computing the first-contact event between each corner of the object *i* and each edge of the object *j*, achieved by equating their respective parametric motions:
  (15) $$\underset{\mathrm{corner}(t)}{\underset{︸}{c_{i,k}\left(0\right)+v_{i}\;t}}\;=\;\underset{\mathrm{edge}(t,u)}{\underset{︸}{a_{j,m}\left(0\right)+v_{j}\;t+u\;e_{j,m}}}\;e_{j,m}=b_{j,m}\left(0\right)-a_{j,m}\left(0\right)$$
  where:
  – Object i∈{ego,target}, and *j* denotes the other object.
  – OBB *i* has four corners $c_{i,k}\left(0\right)$ with k∈{1,2,3,4}.
  – OBB *j* has four edges indexed by m∈{1,2,3,4}, each defined by its endpoints $a_{j,m}\left(0\right)$ and $b_{j,m}\left(0\right)$, and its direction vector $e_{j,m}$.
  A pair (t,u) represents a physically valid collision only if t≥0 and 0≤u≤1. By evaluating all corner–edge interactions, the TTC is defined as the minimum among all valid collision times:
  (16) $$\mathrm{TTC}=\min T\;T=\left\{t_{i,k\to j,m}\;|\;t\geq 0,\;0\leq u\leq 1\right\}$$
  The associated risk metric is the maximum inverse TTC observed over the scenario time interval:
  (17) $$max.TTC^{-1}=\max\left|\frac{1}{TTC(t)}\right|$$
- **Time Exposed to Risk (TET):** This metric quantifies the total duration during which the ego-vehicle is operating under hazardous conditions, defined by a TTC value falling below a critical threshold $TTC^{*}$. It is computed as:
  (18) $$TET=\sum_{t=1}^{T}\gamma_{t}\cdot \Delta t,\;\gamma_{t}=\left\{\begin{array}{cc} 1, & \mathrm{if}\;0<TTC\left(t\right)\leq TTC^{*}\\ 0, & \mathrm{otherwise} \end{array}\right.$$
  where:
  – $\gamma_{t}$ is the switching variable indicating hazardous conditions;
  – $TTC_{j}\left(t\right)$ is the TTC for vehicle *j* at time step *t*;
  – $TTC^{*}$ is the critical TTC threshold;
  – Δt is the simulation time step;
  – *T* is the total number of simulation steps.
  Note: In all experiments, the critical threshold was set to $TTC^{*}=2s$, which corresponds to typical low-speed urban scenarios (<30 km/h) considered in our study (see Section 5.1).

(b) Comfort Metrics

These metrics evaluate the ride smoothness and aim to avoid abrupt maneuvers that may cause discomfort to passengers.

- **Maximum Jerk:** Jerk is the time derivative of acceleration and is widely used as a measure of driving smoothness. Higher jerk values indicate abrupt changes in motion:
  (19) $$Jerk\left(t\right)=\dot{a}\left(t\right)=\ddot{v}\left(t\right),\;Max.Jerk=\max_{t\in [t_{0},t_{f}]}\left|Jerk(t)\right|$$
  where a(t) is the longitudinal acceleration.
- **Maximum Yaw Rate:** The yaw rate quantifies the rate of rotation around the vehicle’s vertical axis and reflects the aggressiveness of turning maneuvers:
  (20) $$YawRate\left(t\right)=\dot{\psi }\left(t\right),\;Max.YawRate=\max_{t\in [t_{0},t_{f}]}\left|\dot{\psi }\left(t\right)\right|$$
  where ψ(t) is the yaw angle at time *t*.

(c) Driving Efficiency Metrics

These metrics evaluate the ability of the ADS to efficiently and accurately complete the driving task:

- **Task Completion Time:** This metric records the total time required by the ego-vehicle to complete the predefined scenario, from the starting zone to the goal. It reflects the temporal efficiency of the system.
- **Lateral Root Mean Square Error (Lateral RMSE):** The lateral RMSE quantifies the average deviation of the ego-vehicle from the centerline of the target lane over time. It is defined as:
  (21) $$\mathrm{Lateral}\;\mathrm{RMSE}=\sqrt{\frac{1}{N}\sum_{i=1}^{N}{\left(e_{i}\right)}^{2}}$$
  where $e_{i}$ is the lateral distance between the ego-vehicle’s position and the lane center at time step *i*. Lower values indicate better trajectory tracking performance.

Overall, these metrics offer a comprehensive and quantifiable assessment of the ADS behavior, facilitating cross-stage and cross-domain performance comparisons. They also provide essential insights for the iterative tuning of both the system parameters (*P*) and the simulation settings (*S*), ultimately supporting a more robust and transferable autonomous driving architecture.

### 5.3. The Results: Performance Comparison Across the Stages

We evaluated the proposed MCRPG methodology across the three stages—DT-Stg, PE-Stg, and RW-Stg—through ten iterative validation loops. For clarity, we report results from the 1st, 5th, and 10th iterations, which sufficiently illustrate the overall convergence trends. Convergence is observed through the Reality Alignment metrics (MNCC-based), which progressively approach values close to 1.0, while the Ego-Vehicle Performance metrics remain consistent across stages. This behavior indicates that both the RG and the PG converge stably toward low values.

The number of iterations required to reach convergence depends on several factors, including the complexity of the ADS, the fidelity of the simulator, the scenario, and the agent models. Moreover, MCRPG has a high sensitivity to initial state: if the parameters are poorly tuned initially, faster convergence may be observed in early iterations, but achieving further improvements in later stages becomes more difficult.

The two-level metric suite guided the iterative tuning of both Simulator and ADS parameters (*S* and *P*), using the logged actions and observations from each run.

Table 2 summarizes the parameters reported in this work and their definitions. In each validation loop, these parameters were updated by the coordinate-search tuning routine, which explored small discrete grids for the most influential parameters listed in Table 2. Although an initial configuration was defined, early validation loops revealed limitations in several assumptions, making it necessary to introduce additional parameters. For example, the analysis of the throttle response in the first iteration revealed that the TABBY EVO presented a noticeable throttle lag, which was not modeled in the simulator. To reduce this RG, we introduced an explicit throttle delay in the simulation and then refined its value over subsequent iterations until it matched the real-world behavior.

These parameters were progressively refined along the validation loops, and Table 3 summarizes the most influential adjustments for each stage—across the 1st, 5th, and 10th iterations. In the first iteration, for example, the maximum speed used by the tracking controller (LQR_v_max), was set to 7.0 m/s in DT-Stg—initially acceptable in simulation. However, during PE-Stg validation this value was reduced to 5.5 m/s to increase the perceived safety during driving sessions in the real vehicle. Scenario parameters were then updated accordingly.

In the following iterations, the focus shifted toward aligning the ego-vehicle’s kinematic response. This was done by tuning simulator parameters related to actuators and powertrain dynamics—such as mass, moment of inertia, throttle/brake gains, and more. Actuator behavior was modeled more realistically by introducing throttle delay and retuning throttle/brake gains, while the effective inertia of the ego was increased by setting the simulated mass to 8500 kg. Although this value did not match the real platform, it compensated for modeling inaccuracies and produced dynamics that better aligned with the real vehicle. In parallel, the tracking controller (LQR) was repeatedly tuned to maintain a balance between minimizing lateral RMSE while ensuring safe and comfortable driving.

Once the longitudinal and lateral dynamics were reasonably aligned across the stages, the tuning process focused on perception. Running processes across multiple ECUs introduced timing delays that were not present in DT-Stg. To mitigate these hidden latencies, perception processes were regrouped to use fewer ECUs, as will be explained later. Additionally, LiDAR cropping parameters (LIDAR_x_max, LIDAR_abs_y_max) were tightened, and the FUSION_min_dist parameter was increased to improve object detection timing across stages. These refinements significantly improved object detection consistency, reducing mismatches that propagated through the entire system stack.

The quantitative results reported in Table 4 show a clear convergence trend across the validation loops. For the Reality Alignment metrics, MNCC values improved significantly, indicating better signal alignment between simulation, parallel execution and real-world data. Our objective with MCRPG—and what Table 4 confirms we achieve—is to increase MNCC values toward 1.0 (i.e., identical signals) for the three stages within the same iteration, while keeping Ego-Vehicle Performance metrics low (lower values indicate better performance). Equally important to optimizing these metrics is obtaining consistent values across stages, since this indicates that the behavior remains coherent in DT-Stg, PE-Stg and RW-Stg—therefore reducing both RG and PG.

Focusing on the Reality Alignment metrics, for example in PE-Stg, the MNCC for the ego-vehicle’s X-position improves from 0.8386 in the first iteration to 0.9972 in the final validation, indicating near-perfect alignment. Moreover, by the 10th iteration, nearly identical MNCC values are achieved across the three stages. Similar improvements are observed in steering and velocity. Object detection alignment also improved along both axes, particularly along the y-axis, where the MNCC increases from 0.6905 to 0.9591 in PE-Stg.

In terms of Ego-Vehicle Performance, a clear PG was observed in the first iteration due to the RG, the decreased LQR_v_max, and the fact that the *P* parameters were tuned solely to maximize performance in DT-Stg. At that initial stage, many simulator settings (*S*) were left at their defaults, and real-world constraints were not yet considered. Once real-world feedback became available from the second iteration onward–and with parameter fine-tuning—in the latest iterations significant improvements were achieved in safety, comfort, and driving-efficiency metrics.

Safety indicators confirm the benefits of more conservative control and more accurate actuator modeling. From very high risk exposures during the first loop in PE-Stg/RW-Stg (Max. TTC^−1^ = 93.44/8.85 s^−1^; TET = 6.71/3.58 s), both metrics drop to near-zero in the 10th iteration (Max. TTC^−1^ = 0.38/0.41 s^−1^; TET = 0.00/0.00 s). Indicating that the adjusted simulated dynamics no longer lead to driving into risky situations.

Throughout the loops, PE-Stg served as an effective bridge: it revealed safety deficits in the early stages—where TTC^−1^ and TET metrics were high—during real-vehicle driving sessions while keeping perception agents virtual. This allowed us to anticipate these risk scenarios without exposing the system to real-world danger or the logistical challenges of deploying real adversaries. At the same time, it ensured RW-Stg consistent performance once the system was prepared for risk-free validations.

Comfort metrics evolve in parallel. In the first simulation, we observe a maximum jerk value of 5.0593 m/s^3^, reflecting abrupt accelerations and steering corrections. This level of jerk is neither safe nor comfortable, and the issue was not initially detected in the simulator. Once this behavior was identified in the real vehicle—during subsequent stages—the LQR and low-level control parameters were tuned. As a result, jerk was progressively reduced and became more stable across stages, leading to smoother trajectories.

Driving efficiency was also affected by these changes. Safety improvements came at the cost of longer completion times: in DT-Stg, task completion time increases from 83.305 s in the first loop to 107.02 s in the last iteration, reflecting a more conservative behavior. Nonetheless, lateral tracking improved across all stages, with Lateral RMSE values decreasing from 1.0592 m to 0.7084 m for RW-Stg, confirming the effectiveness of the tracking controller tuning.

**Figure 12:**
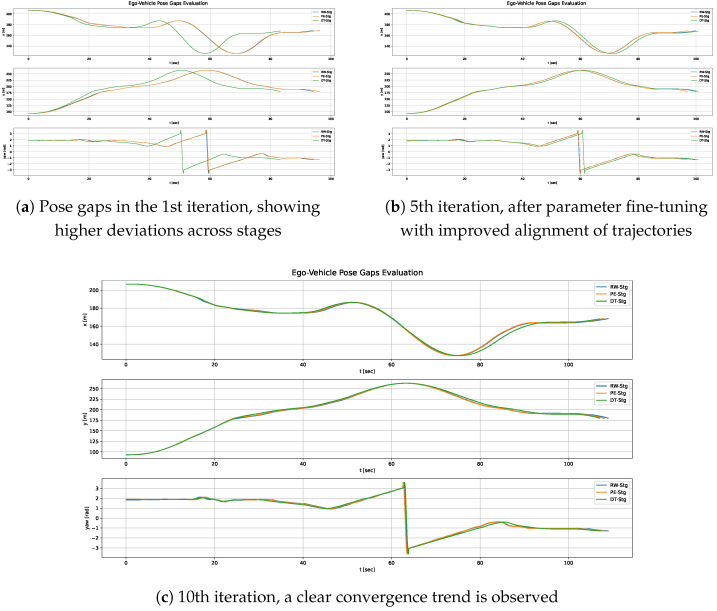
Ego-vehicle pose gaps over iterations.

**Figure 13:**
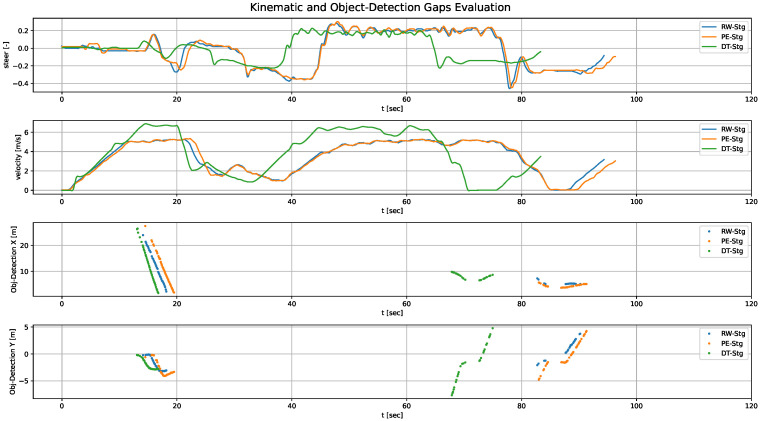
Kinematic and object-detection signals across stages for the 1st iteration.

**Figure 14:**
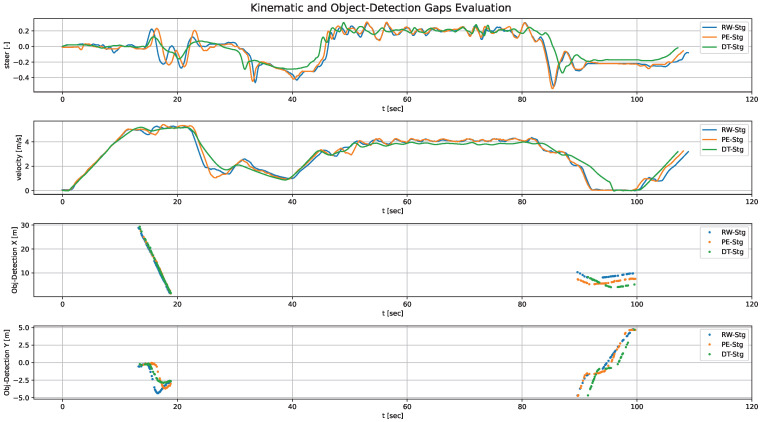
Kinematic and object-detection signals for the 10th iteration, showing the convergence across the stages.

Figure 12, Figure 13 and Figure 14 provide a graphical view of the gaps shown in Table 4, highlighting how signals converge across iterations. Figure 12a illustrates the ego-vehicle pose (x, y, and yaw) in the 1st iteration. It revealed noticeable discrepancies between the three stages, particularly between DT-Stg and PE-Stg. This is mainly due to the fact that, in the 2nd stage of that iteration, LQR_v_max was modified, resulting in a PG gap. In the second iteration the situation improved substantially, but we continued to observe discrepancies because the ego’s kinematic response was not yet properly modeled. Subsequent iterations refined this behavior.

In Figure 12b we see how the RG improved significantly once the ego’s kinematic response was adjusted, although perception-related gaps were still present. From this point on, the focus shifted to reducing perception gaps, ultimately achieving the behavior shown in Figure 12c. This final iteration shows the gaps greatly reduced, with the trajectories across stages nearly overlapping, demonstrating the effectiveness of the methodology.

Although Figure 12 shows only the ego-vehicle’s position, similar discrepancies were observed across all other signals during the iterative process. It is important to emphasize that these observations must be analyzed using time-dependent signals. For instance, plotting the ego-vehicle position in the x-y plane without considering the temporal dimension can lead to misleading conclusions, giving the impression that no gap exists when one actually does: the vehicle may pass through the same spatial points (no observable gap) but at different times.

Therefore, the analysis must account for the temporal evolution of the signals to accurately identify misalignments among them. Figure 13 illustrates the kinematic and object detection signals across stages for the first iteration, while Figure 14 shows the observations corresponding to the 10th iteration, once again demonstrating the convergence trend of the signals throughout the validation loops.

Beyond bridging RG and PG, the methodology revealed system-level issues that neither pure simulation nor real-world testing exposed. For example, during PE-Stg, perception initially ran on a Jetson AGX Orin device receiving simulated sensor data from a secondary PC, introducing hidden latency. While invisible during online driving, this latency was revealed by offline metric analysis. To solve this, we migrated the perception module to run directly on the simulator PC during PE-Stg, which significantly improved signal alignment, especially for object detection.

This example illustrates how our methodology not only bridges RG and PG but also reveals critical SW and HW integration issues. Similar issues related to poorly tuned agent dynamics, time synchronization, code optimization so that the execution time is similar in all stages, sensor misconfigurations, and latency were identified and resolved through the same analytical approach.

In summary, across the three stages and iterative loops, the MCRPG pipeline achieved near-perfect alignment of ego-vehicle’s kinematics and object detection observations, together with consistent improvements in safety and comfort metrics. These results validate the staged MCRPG framework as an effective methodology to progressively close RG and PG in urban scenarios, while simultaneously revealing optimization opportunities and uncovering hidden integration issues that would likely remain unnoticed under conventional simulation or real-world evaluation approaches.

## 6. Conclusions and Future Work

In this work, we present our Methodology for Closing Reality and Performance Gaps (MCRPG), a structured approach designed to jointly reduce both the Reality Gap (RG) and the Performance Gap (PG) during the transfer of an Autonomous Driving Stack (ADS) from simulation to real-world environments. The methodology is organized into three iterative stages—Digital Twin, Parallel Execution, and Real-World—supported by a two-level metric suite (Reality Alignment and Ego-Vehicle Performance) that enables a smooth, measurable, and repeatable transition across domains.

MCRPG was designed from the beginning to be scalable across different architectures, simulators, and Operational Design Domains (ODDs) of increasing complexity. The core approach relies on iterative tuning of ADS parameters (*P*) and simulator settings (*S*) throughout the validation stages (DT-Stg → PE-Stg → RW-Stg), and is therefore applicable to other ODDs with a validation cost that grows proportionally with scenario complexity. In this sense, expanding the ODD (or increasing scenario richness within the same ODDs) can further improve behavior analysis and help to close the Sim2Real gaps, but it also increases the number of parameters to be managed, the complexity of the analysis, the fine-tuning effort, and the overall validation costs.

In parallel, the overall architecture is based on a modular built on ROS and containers, which allows modules to be replaced or added without reworking the platform, supporting efficient scaling to more complex ODD. However, practical scalability remains mainly constrained by the simulator capabilities, real-world infrastructure, available hardware resources, and project cost.

Implemented on an open-source, low-cost Development and Validation Platform (DVP) that integrates CARLA, ROS, and a physical testbed, the proposed methodology provides a practical and replicable Sim2Real workflow applicable to different Autonomous Driving Stacks (ADSs). Across multiple validation loops, the experimental campaign demonstrated a progressive convergence between simulated and real-world behaviors. Reality Alignment metrics, based on MNCC, showed increasingly consistent alignment of kinematic and perception signals over iterations while Ego-Vehicle Performance metrics stabilized around coherent values in terms of safety and comfort.

Within this approach, the PE-Stg proved to be a key component, by synchronizing the real vehicle with its digital twin and enabling the generation and coordination of virtual agents through OpenSCENARIO. This significantly reduced the risk associated with the Real-World stage, enabling these validations once the ADS was ready to face the scenario’s use cases. In addition, PE-Stg plays a central role in scalability by enabling real-world testing without physically deploying all actors, acting as a lever for logistical scalability as the number of agents or scenario diversity increases. For this reason, OpenSCENARIO was selected to define and scale scenarios directly, leveraging the benefits of the standard to increase scenario richness in a controlled and reusable manner.

Beyond reducing gaps, MNCC also acts as an effective diagnostic and redesign tool. The iterative analysis of actions and observations revealed some HW/SW integration issues—hidden latencies, inaccurately modeled actuators, misaligned agent dynamics, and time-synchronization inconsistencies—that were not apparent during online driving but surfaced during offline evaluation. Consequently, the methodology not only supports parameter tuning but also enables continuous refinement of the SW and HW resource allocation, contributing to more robust ADS implementations.

Despite these advantages, some limitations point toward promising future work. Expanding the experimental scope to more challenging scenarios—including high-speed highways, dense urban traffic, adverse weather conditions, tunnels, and more—remains essential. Likewise, the metric suite should be enhanced with additional indicators analyzing perception accuracy, prediction quality, latencies for each module, computational load, and energy consumption.

Another relevant work involves evaluating the effectiveness of MNCC across different platforms, including vehicles with different sensor and HW configurations or other simulators. Furthermore, future work may explore a further automation of the tuning process for both *S* and *P* parameters through more advanced optimization strategies or AI-based methods, beyond the coordinate-search scheme used in this work. Examples include Bayesian optimization and evolutionary search for sample-efficient exploration of high-dimensional parameter spaces, as well as reinforcement-learning-based or other gradient-free policy search techniques tightly coupled with the proposed metric suite.

When combined with the current strengths of our MNCC and the DVP, these future efforts aim to advance toward a fully scalable and robust development and validation framework, capable of serving as a reference methodology for bridging Sim2Real gaps and accelerating the safe deployment of autonomous driving technologies in real-world environments.

## Figures and Tables

**Table 1:** Technical specifications of the TABBY EVO.

Parameter	Value	Parameter	Value
**Length**	3030 mm	**Max. Climb**	25%
**Width**	1488 mm	**Turn Radius**	5 m
**Height**	1380 mm	**Max. Steering Angle**	±20°
**Weight (Batteries incl.)**	730 kg	**Max. Motor Power**	29.5 kW @ 2500 rpm
**Batteries Weight**	140 kg	**Max. Motor Torque**	128 N·m @ 500 rpm
**Wheelbase**	2360 mm	**Max. Motor RPM**	5500 rpm
**Track Width**	1315 mm	**Rated Voltage**	80 Vac
**Top Speed**	120 km/h	**Max. Current**	400 A
**Nominal Range**	100 km	**Max. Power Factor**	0.97
**Tires**	175/55R15	**Reduction Gearbox Ratio**	5.8:1

**Table 2:** Parameters definition.

Parameters Set	Parameter	Description
*P*	LQR_v_max (m/s)	Maximum speed allowed for tracking controller.
	LQR_rc_max (m)	Max allowed radius of curvature, inversely related to speeds while cornering.
	LQR_q11 (−)	Weight on lateral error, higher values keep the ego closer to the path but may increase oscillations.
	LQR_q22 (−)	Weight on heading error, higher values allow faster alignment and reduce straight-line oscillations.
	LQR_r11 (−)	Weight on steering-effort changes, higher values produce smoother/slower steering responses.
	MPC_w (m)	Lateral offset used by the MPC to avoid obstacles.
	DECISION_PC_d_max (m)	Distance used to calculate Pedestrian Crossing velocities.
	DECISION_PC_d_min (m)	Distance at which the ego must stop if the Pedestrian Crossing is occupied.
	DECISION_OT_d_max (m)	Distance at which the overtaking maneuver can start.
	DECISION_OT_speed (m/s)	Speed at which the overtaking starts.
	FUSION_min_dist (m)	Minimum matching distance for camera–LiDAR fusion.
	LIDAR_x_max (m)	Longitudinal cropping limit of the LiDAR point cloud in front of the ego.
	LIDAR_abs_y_max (m)	Lateral cropping limit of the LiDAR point cloud on both sides.
*S*	PEDESTRIAN_speed (m/s)	Pedestrian speed used in scenario definition.
	EGO_mass (kg)	Ego-vehicle mass in the CARLA simulator.
	EGO_max_steer_angle (°)	Maximum steering angle of the front wheels.
	EGO_dr_full_throttle (−)	Internal resistance of the engine to acceleration. Low values indicate rapid acceleration (e.g., sports or electric vehicles).
	EGO_dr_zero_throttle (−)	Engine deceleration rate when the accelerator is released.
	EGO_moi (−)	Parameter affecting how quickly engine RPM increases or decreases.
	THROTTLE_kp (−)	Proportional gain of the throttle controller in CARLA; higher values yield a more reactive response but may cause overshoot.
	THROTTLE_ki (−)	Integral gain of the throttle controller; helps eliminate steady-state speed error.
	THROTTLE_delay (sec)	Delay implemented to match real-world behavior; TABBY EVO presents a default throttle lag.
	BRAKE_kp (−)	Proportional gain of the brake controller.
	BRAKE_ki (−)	Integral gain of the brake controller.
	GNSS_x (m)	Longitudinal position of the GNSS relative to the ego’s geometric center.
	CAMERA_x (m)	Longitudinal position of the Camera relative to the ego’s geometric center.

**Table 3:** Parameters fine tuning across the iterations.

Parameters Set	Parameter	1st Iteration				5th Iteration				10th Iteration		
		DT-Stg	PE-Stg	RW-Stg		DT-Stg	PE-Stg	RW-Stg		DT-Stg	PE-Stg	RW-Stg
*P*	LQR_v_max (m/s)	7.0000	5.5000	5.5000	…	5.5000	5.5000	5.5000	…	5.5000	5.5000	5.5000
	LQR_rc_max (m)	25.000	25.000	25.000		30.000	30.000	30.000		40.000	40.000	40.000
	LQR_q11 (−)	20.000	20.000	20.000		30.000	30.000	30.000		30.000	30.000	30.000
	LQR_q22 (−)	100.00	10.000	10.000		1.0000	1.0000	1.0000		0.0001	0.0001	0.0001
	LQR_r11 (−)	9000.0	5000.0	5000.0		5000.0	5000.0	5000.0		5000.0	5000.0	5000.0
	MPC_w (m)	3.5000	2.7500	2.7500		2.7500	2.7500	2.7500		2.7500	2.7500	2.7500
	DECISION_PC_d_max (m)	30.000	30.000	30.000		30.000	30.000	30.000		40.000	40.000	40.000
	DECISION_PC_d_min (m)	10.000	10.000	10.000		10.000	15.000	15.000		15.000	15.000	15.000
	DECISION_OT_d_max (m)	40.000	40.000	40.000		40.000	30.000	30.000		30.000	30.000	30.000
	DECISION_OT_speed (m/s)	7.0000	7.0000	7.0000		7.0000	5.0000	5.0000		5.0000	5.0000	5.0000
	FUSION_min_dist (m)	2.5000	2.5000	2.5000		2.5000	2.5000	4.0000		4.5000	4.5000	4.5000
	LIDAR_x_max (m)	50.000	50.000	50.000		50.000	50.000	30.000		30.000	30.000	30.000
	LIDAR_abs_y_max (m)	10.000	10.000	10.000		10.000	10.0000	10.000		5.0000	5.0000	5.0000
*S*	PEDESTRIAN_speed (m/s)	3.0000	1.7500	1.7500		1.7500	1.7500	1.7500		1.7500	1.7500	1.7500
	EGO_mass (kg)	1000.0	1000.0	1000.0		4000.0	4000.0	4000.0		8500.0	8500.0	8500.0
	EGO_max_steer_angle (°)	25.000	25.000	25.000		25.000	25.000	25.000		20.000	20.000	20.000
	EGO_dr_full_throttle (−)	0.1500	0.1500	0.1500		0.0500	0.0500	0.0500		0.0500	0.0500	0.0500
	EGO_dr_zero_throttle (−)	0.1000	0.1000	0.1000		0.5000	0.5000	0.5000		0.5000	0.5000	0.5000
	EGO_moi (−)	1.0000	1.0000	1.0000		0.7500	0.7500	0.7500		0.7500	0.7500	0.7500
	THROTTLE_kp (−)	0.5000	0.5000	0.5000		1.2500	1.2500	1.2500		1.2500	1.2500	1.2500
	THROTTLE_ki (−)	0.0250	0.0250	0.0250		0.0050	0.0050	0.0050		0.0050	0.0050	0.0050
	THROTTLE_delay (sec)	-	-	-		0.1500	0.1500	0.1500		0.1500	0.1500	0.1500
	BRAKE_kp (−)	1.0000	1.0000	1.0000		0.0900	0.0900	0.0900		0.0900	0.0900	0.0900
	BRAKE_ki (−)	0.0000	0.0000	0.0000		0.0005	0.0005	0.0005		0.0005	0.0005	0.0005
	GNSS_x (m)	−0.600	−0.600	−0.600		−0.600	−0.600	−0.600		−1.000	−1.000	−1.000
	CAMERA_x (m)	0.3750	0.3750	0.3750		0.3750	0.3750	0.3750		0.4000	0.4000	0.4000

**Table 4:** Results across iterative loops: Reality Alignment and Ego-Vehicle Performance.

Aspect Group	Metric	1st Iteration				5th Iteration				10th Iteration			Objective
		DT-Stg	PE-Stg	RW-Stg		DT-Stg	PE-Stg	RW-Stg		DT-Stg	PE-Stg	RW-Stg	
Reality Alignment (MNCC)	Ego-Vehicle X	-	0.8386	0.9861	…	0.9806	0.9904	0.9997	…	0.9970	0.9972	**0.9998**	↑ (≈1.0) and consistent across stages
	Ego-Vehicle Y	-	0.9026	0.9798		0.9948	0.9973	0.9997		0.9991	0.9994	**0.9998**	
	Steer	-	0.7235	0.9689		0.9280	0.9425	0.9799		**0.9834**	0.8915	0.9604	
	Velocity	-	0.5836	0.9877		0.9332	0.9580	0.9855		0.9568	0.9655	**0.9893**	
	Obj-Detection X	-	0.9403	0.9687		0.8058	0.8886	0.9897		0.9847	**0.9962**	0.9940	
	Obj-Detection Y	-	0.6905	0.8784		0.6372	0.8622	0.9525		0.9671	0.9591	**0.9808**	
Ego-Vehicle Performance	Max. TTC^−1^ (s^−1^)	0.6633	93.441	8.8570		0.7347	33.118	0.5614		**0.3778**	0.3847	0.4147	↓ and consistent across stages
	TET (s)	0.6947	6.7158	3.5821		1.7869	6.5243	0.7759		**0.0000**	0.0000	0.0000	
	Max. Jerk (m/s^3^)	5.0593	2.2039	2.4506		2.6059	4.1848	4.0026		2.7441	2.4643	**1.8712**	
	Max. Yaw Rate (rad/s)	1.6192	1.6637	1.6505		1.6009	1.6644	**1.5199**		1.5978	1.6084	1.5623	
	Task Completion Time (s)	**83.305**	96.323	94.294		100.73	96.976	99.862		107.02	108.02	108.94	
	Lateral RMSE (m)	0.8022	1.1165	1.0592		0.6685	0.8511	0.9042		**0.5791**	0.6790	0.7084	

## Data Availability

The original contributions presented in this study are included in the article. Further inquiries can be directed to the corresponding author.
